# Supplementary material for: Splice-Junction-Based Mapping of Alternative Isoforms in the Human Proteome
Source: Cell Rep. Author manuscript; Available in PMC 2020 Jan 15. (PMC6961840; doi:10.1016/j.celrep.2019.11.026)
Supplement: 2 [file NIHMS1546469-supplement-2.zip › DF1/PXD010154/Esophagus/Esophagus_13_NEXN_QEMGEEDDVDVRPAR.pdf]

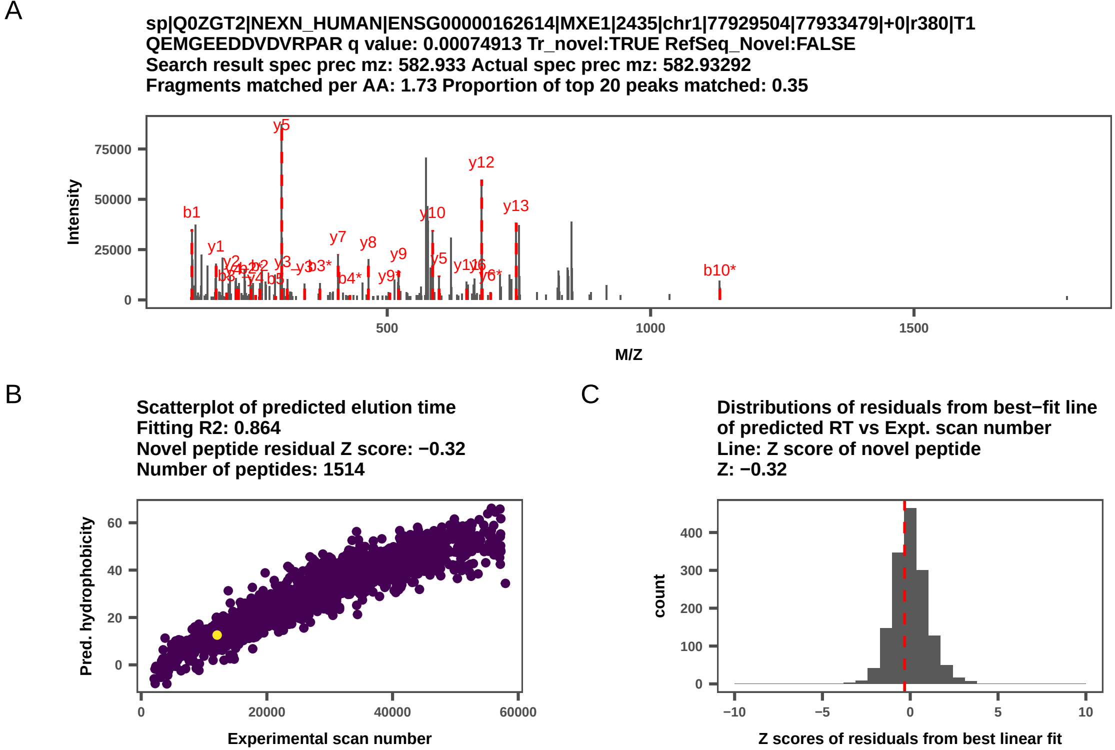
A
sp|Q0ZGT2|NEXN_HUMAN|ENSG00000162614|MXE1|2435|chr1|77929504|77933479|+0|r380|T1
QEMGEEDDVDVRPAR q value: 0.00074913 Tr_novel:TRUE RefSeq_Novel:FALSE
Search result spec prec mz: 582.933 Actual spec prec mz: 582.93292
Fragments matched per AA: 1.73 Proportion of top 20 peaks matched: 0.35
Intensity
75000
50000
25000
0
500
1000
1500
M/Z
y5
b1
y1
y12
y10
y13
y7
y8
y9
y3
b3*
b4*
y9*
y6*
b5
b10*
B
Scatterplot of predicted elution time
Fitting R2: 0.864
Novel peptide residual Z score: −0.32
Number of peptides: 1514
Pred. hydrophobicity
60
40
20
0
0
20000
40000
60000
Experimental scan number
C
Distributions of residuals from best−fit line
of predicted RT vs Expt. scan number
Line: Z score of novel peptide
Z: −0.32
count
400
300
200
100
0
−10
−5
0
5
10
Z scores of residuals from best linear fit
